# Editorial Comment: A modified clinicopathological tumor staging system for survival prediction of patients with penile cancer

**DOI:** 10.1590/S1677-5538.IBJU.2020.02.06

**Published:** 2020-01-10

**Authors:** Gustavo Cardoso Guimarães

**Affiliations:** 1 Departamento de Oncologia Cirúrgica Beneficência Portuguesa de São Paulo São PauloSP Brasil Chefe do Departamento de Oncologia Cirúrgica, Beneficência Portuguesa de São Paulo, São Paulo, SP, Brasil

Li ZS ^1,2,3,4^, Ornellas AA ^5^, Schwentner C ^6^, Li X ^7^, Chaux A ^8^, Netto G ^9^, Burnett AL ^10^, Tang Y ^11^, Geng J ^12^, Yao K ^2,3,4^, Chen XF ^13^, Wang B ^14^, Liao H ^15^, Liu N ^16^, Chen P ^17^, Lei YH ^18^, Mi QW ^19^, Rao HL ^20^, Xiao YM ^15^, Wang QL ^18^, Qin ZK ^2,3,4^, Liu ZW ^2,3,4^, Li YH ^2,3,4^, Zou ZJ ^7^, Luo JH ^21^, Li H ^22^, Han H ^23,24,25^, Zhou FJ ^26,27,28^

^1^ Department of Urology, Shenzhen People’s Hospital, The Second Clinical College of Jinan University, Shenzhen, Guangdong, P. R. China; ^2^ Sun Yat-sen University Cancer Center; State Key Laboratory of Oncology in South China; Collaborative Innovation Center of Cancer Medicine, Guangzhou, Guangdong, P. R. China; ^3^ Department of Urology, Sun Yat-sen University Cancer Center, Guangdong, P. R. China; ^4^ Department of Urology, Brazilian National Institute of Cancer and Hospital Mário Kröeff, Rio de Janeiro, Brazil; ^5^ Section of Urology, National Institute of Cancer, Rio de Janeiro, Brazil; ^6^ Department of Urology, Diakonie Klinikum Stuttgart, Stuttgart, Germany; ^7^ Urological Department, Urological Institute, West China Hospital of Sichuan University, Chengdu, Sichuan, P. R. China; ^8^ Department of Scientific Research, Norte University, Asunción, Paraguay; ^9^ Department of Pathology, Johns Hopkins University, Baltimore, MD, USA; ^10^ Department of Urology, Johns Hopkins University, Baltimore, MD, USA; ^11^ Department of Urology, Affiliated Cancer Hospital of Guangxi Medical University, Nanning, Guangxi, P. R. China; ^12^ Department of Urology, Kaohsiung Medical University Hospital, Kaohsiung, Taiwan, China; ^13^ Department of Urology, The First People’s Hospital of Chenzhou, Chenzhou, Hunan, P. R. China; ^14^ Department of Urology, Cancer Center of Guangzhou Medical University, Guangzhou, Guangdong, P. R. China; ^15^ Department of Urology, Sichuan Cancer Hospital, Chengdu, Sichuan, P. R. China; ^16^ Department of Urology Oncological Surgery, Chongqing Cancer Hospital & Institute & Cancer Center, Chongqing, China; ^17^ Department of Urology, Affiliated Tumor Hospital of Xinjiang Medical University, Urumqi, Xinjiang, P. R. China; ^18^ Department of Urology, Yunnan Provincial Tumor Hospital, The Third Affiliated Hospital of Kunming Medical University, Kunming, Yunnan, P. R. China; ^19^ Department of Urology, Dongguan People’s Hospital, Dongguan, Guangdong, P. R. China; ^20^ Department of Pathology, Sun Yat-sen University Cancer Center, Guangzhou, 510060, Guangdong, P. R. China; ^21^ Department of Urology, First Affiliated Hospital, Sun Yat-sen University, Guangzhou, Guangdong, P. R. China; ^22^ Department of Pathology, The Chinese University of Hong Kong, Hong Kong, P. R. China; ^23^ Sun Yat-sen University Cancer Center; State Key Laboratory of Oncology in South China; Collaborative Innovation Center of Cancer Medicine, Guangzhou, Guangdong, P. R. China; ^24^ Department of Urology, Sun Yat-sen University Cancer Center, Guangdong, P. R. China; ^25^ Department of Urology, Brazilian National Institute of Cancer and Hospital Mário Kröeff, Rio de Janeiro, Brazil; ^26^ Sun Yat-sen University Cancer Center; State Key Laboratory of Oncology in South China; Collaborative Innovation Center of Cancer Medicine, Guangzhou, Guangdong, P. R. China; ^27^ Department of Urology, Sun Yat-sen University Cancer Center, East Guangzhou, Guangdong, P. R. China; ^28^ Department of Urology, Brazilian National Institute of Cancer and Hospital Mário Kröeff, Rio de Janeiro, Brazil

Cancer Commun (Lond). 2018 Nov 23;38(1):68

**DOI:** 10.1186/s40880-018-0340-x | **ACCESS:** 10.1186/s40880-018-0340-x

## COMMENT

In this interesting paper, Drs. Zai-Shang Li, Antonio Augusto Ornellas and colleagues, test the prognostic validity of the The 8th American Joint Committee on Cancer tumor–node–metastasis (AJCC-TNM) staging system and to determine whether a modified clinicopathological tumor staging system that includes lymphovascular embolization could increase the accuracy of prognostic prediction for patients with stage T2–3 penile cancer.

The presence of lymphovascular embolization, perineural invasion, and the degree of differentiation are all considered prognostic indicators of survival for penile cancer patients (1, 2)

They analyzed 411 patients who were treated at 2 centers (China and Brazil) between 2000 and 2015. They were staged according to the 8th AJCC-TNM staging system. The internal validation was analyzed by bootstrap-corrected C-indexes and to external validation, where used the data from 436 patients treated at 15 centers over four continents.

The authors found a survivorship overlap was observed between T2 and T3 patients classified according to the 8th AJCC-TNM staging system. The T2 and T3 patients with lymphovascular embolization showed significantly shorter CSS than did those without lymphovascular embolization (P < 0.001).

The authors proposed a modifications to the 8th AJCC-TNM staging system with the T2 and T3 categories should be subdivided into two new categories as follows: t2 tumors invade the corpus spongiosum and/or corpora cavernosa and/or urethra without lymphovascular invasion, and t3 tumors invade the corpus spongiosum and/or corpora cavernosa and/or urethra with lymphovascular invasion.

With this modifications they suggest that the new staging system which the involving lymphovascular embolization showed improved prognostic stratification with significant differences in CSS among all categories (all P < 0.005) and exhibited higher accuracy in predicting patient prognoses than did the 8th AJCC-TNM staging system (C-index, 0.739 vs. 0.696). And they show that these results were confirmed in the external validation cohort.

Further studies with other patient cohorts should be performed to validate these findings.

## References

[1] 1. Guimarães GC, Cunha IW, Soares FA, Lopes A, Torres J, Chaux A, et al. Penile squamous cell carcinoma clinicopathological features, nodal metastasis and outcome in 333 cases. J Urol. 2009;182:528-34; discussion 534. 10.1016/j.juro.2009.04.028 19524964

[2] 2. Emerson RE, Ulbright TM, Eble JN, Geary WA, Eckert GJ, Cheng L. Predicting cancer progression in patients with penile squamous cell carcinoma: the importance of depth of invasion and vascular invasion. Mod Pathol. 2001;14:963-8. 10.1038/modpathol.3880419 11598165

